# Physiotherapy Interventions for Preventing Spinal Curve Progression in Adolescent Idiopathic Scoliosis: A Systematic Review

**DOI:** 10.7759/cureus.30314

**Published:** 2022-10-14

**Authors:** Tabish Fahim, Sakshi Virsanikar, Diya Mangharamani, Sabih N Khan, Shrikant Mhase, Laxmikant Umate

**Affiliations:** 1 Department of Physiotherapy, Mahatma Gandhi Mission (MGM) School of Physiotherapy, Aurangabad, IND; 2 Department of Rehabilitation, Mahatma Gandhi Mission (MGM) School of Physiotherapy, Aurangabad, IND; 3 Department of Cardiorespiratory Physiotherapy, Mahatma Gandhi Mission (MGM) School of Physiotherapy, Aurangabad, IND; 4 Department of Community Physiotherapy, Mahatma Gandhi Mission (MGM) School of Physiotherapy, Aurangabad, IND; 5 Department of Research and Development, Datta Meghe Institute of Medical Sciences, Wardha, IND

**Keywords:** scopus, pedro, pubmed, systematic review, cobb angle, exercises, physiotherapy, idiopathic adolescence scoliosis

## Abstract

Adolescent idiopathic scoliosis* *(AIS) is an abnormal curvature of the spine that appears in late childhood or adolescence. The aim of this systematic review was to present and synthesize the most relevant therapeutic advice and evidence on the efficacy of physiotherapy exercises for preventing the growth of spinal curvature caused by adolescent idiopathic scoliosis. "Adolescent idiopathic scoliosis," "exercise," "Cobb angle," and "physiotherapy" were the sole keywords used for the published research. Using these keywords and a combination of them, electronic resources such as PubMed, Cochrane Central Register of Controlled Trials, Physiotherapy Evidence Database (PEDro), Elton B. Stephens Company (EBSCO) host, and ScienceDirect (Elsevier) were searched. The search was restricted to studies that were conducted in English between 2010 and 15 January 2022 that were controlled, randomized, and non-randomized. Studies were selected based on their titles and abstracts, with the exception of any that did not pertain to the study's goals. The Cobb angle was the important outcome measure. For each intervention, the Cobb angle's mean change score, the difference between the final and baseline scores, was determined. Nine studies were evaluated to be of outstanding quality out of a total of 20 studies that were reviewed for eligibility. With an exercise regimen of at least seven weeks, controls on lowering the Cobb angle in patients with AIS would provide encouraging outcomes. It also shows that bracing can strengthen the Cobb angle compared to exercise in the community. However, long-term orthotic activity ultimately results in trunk resistance and muscle loss in the center of the back. The combination of techniques and treatment methods seems to have better results in treating scoliosis, particularly using exercises involving the Schroth and scientific exercise approach to scoliosis (SEAS).

## Introduction and background

Scoliosis is a three-dimensional spine deformity characterized by a greater than 10-degree lateral curvature of the spine in the coronal plane [1]. It is the most common spinal abnormality and gets its name from the ancient Greek word "skolios," which means "curved" [2,3]. It can be classified into different categories according to the age of onset, etiology, incidence, and curve shape; for example, it can be grouped according to causes into three main types: congenital, syndromic, and idiopathic [1,3,4]. Congenital scoliosis is a spinal deformity caused by the failure of normal vertebral development, whereas syndromic scoliosis is caused by neurofibromatosis, other significant medical problems, or the dysfunction of the neuromuscular, skeletal, and connective tissue systems. Idiopathic scoliosis has no known etiology and can be categorized into the following categories according to the patient's age at the time of diagnosis: infantile idiopathic scoliosis (IIS), juvenile idiopathic scoliosis (JIS), and adolescent idiopathic scoliosis (AIS) [5]. IIS affects patients mainly in the age group of three years and below, JIS affects ages between three and nine years, and AIS affects patients aged 10-18 years. The general term AIS means that adolescents aged 10 years or more are diagnosed with scoliosis, and idiopathic means that the definitive etiology and etiopathogenesis remain unknown. Thoracolumbar/lumbar curves are most common in males; however, females have a higher percentage of thoracic and double curves, but the etiology and pathogenesis of this condition remain unclear. Generally, the patient, family, general practitioner, or school nurse notes postural changes first [6]. However, a detailed medical history, physical examination, and standard scoliosis radiographs have clarified the diagnosis of AIS.

The Cobb angle is the curvature of the spine, and measuring it is essential for determining the severity of scoliosis, selecting the best course of action, and monitoring the progression or regression of cases following treatment [7,8]. There are many ways to measure and calculate the Cobb angle; for example, it can be performed manually by measuring the spinal angle on an X-ray posterior-anterior (PA) film, which is the standard approach used to measure the scoliosis angle by identifying the upper and lower vertebrae of the spinal deformity, drawing lines extending along vertebral borders, and measuring the Cobb angle directly or geometrically, digitally using a smart phone, radiographic program, or others [9]. Curves measuring less than 25 degrees were classified as mild, those measuring 25-40 degrees were moderate, and more than 40 degrees were considered severe. The angle of trunk rotation and the apex of the curve deformity are measured using a scoliometer or inclinometer in which an angle of five degrees or less is considered normal whereas an angle of seven or more is considered abnormal [10]. It is impossible to stop the event of scoliosis; therefore, early detection is currently supported to ensure that appropriate treatment is often provided. The utilization of forward-bending screening tests may be a controversial issue, but it is impossible to avoid scoliosis; therefore, "preventive" interventions are limited to early intervention and prompt therapy [11].

These two different approaches to management (non-surgical versus surgical) seem common in various parts of the world [12]. The wait-and-see strategy is common in the United States, the United Kingdom, and Australia, but exercises and bracing are widely recommended for patients in different areas of Europe [13]. The goal of non-surgical treatment during adolescence is to stop curve advancement, whereas curve correction and maintenance are the objectives of surgical intervention. However, pulmonary function is the only negative consequence of AIS and is strongly correlated with curve size. Therefore, when selecting a treatment plan, therapists must be aware of the risk of curve progression. The primary objective of non-surgical treatment is to restrict the number of surgical interventions by reducing curve progression [14]. For the non-surgical management of AIS, a physiotherapy approach that is increasingly widespread involves physical exercises, rehabilitation programs, and the use of braces, which are commonly used to treat AIS [15,16]. However, the efficacy of brace therapy remains controversial [17]. The main goal of AIS bracing is to avoid or prevent the development of spinal deformity curves until skeletal maturity occurs during growth [16]. Bracing is frequently used for patients with spinal curves between 25 and 45 degrees who are skeletally immature. Nevertheless, if a patient has a high probability of curve advancement and the curve is less than 25 degrees, it can be employed [18]. In patients with AIS, bracing substantially decreases the development of high-risk curves to meet the requirements of surgical procedures, and this advantage increases with prolonged periods of brace use [14,19].

However, in order to improve strength, spinal mobility, balance, and spinal deformity in AIS, physical scoliosis exercises must be started as the first-line treatment for mild scoliosis in patients with a low risk of curve advancement [20,21]. Various physiotherapy interventions are available to treat AIS, such as Schroth physiotherapy scoliosis-specific exercises (PSSE), core stabilization (CS) exercises, stretching and massage, and manual techniques. This literature review indicates that there is no research that examines how different physical activities affect the Cobb angle in AIS. Therefore, the primary aim of this research is mainly to discuss and compare which physical exercises can have effect on the Cobb angle, and the secondary aim is to check the efficacy of bracing in preventing spinal curvature in AIS.

## Review

Research methodology

Single keywords were used to search the electronic databases PubMed, Cochrane Central Register of Controlled Trials, Physiotherapy Evidence Database (PEDro), Elton B. Stephens Company (EBSCO) host, and ScienceDirect (Elsevier). In order to conduct the process, Preferred Reporting Items for Systematic Reviews and Meta-Analyses for Searching (PRISMA-S) guidelines were followed, and the search items included "adolescent idiopathic scoliosis," "exercise," "Cobb angle," and "physiotherapy," as well as a combination of keywords. The controlled, randomized, and non-randomized trials conducted in the English language between 2010 and 15 January 2022 were only considered in the bibliographical search. The future inaccessible article authors were contacted and asked to include the full text of their publications. With the exception of those that did not relate to the objectives of this study, the publications were chosen based on their titles and abstracts, and consensus among the reviewers was reached through debate. The search strategies used for the selected studies are listed in Table 1.

**Table 1:** Search strategies used for the selected studies. PEDro: Physiotherapy Evidence Database; EBSCOhost: Elton B. Stephens Company host; #: hashtag.

Database/date	Searches	Keyword; ALL Fields; TX All Text; Title or Abstract
Cochrane Central Register of Controlled Trials/15 January 2022	#1	Keyword (adolescent idiopathic scoliosis)
	#2	Keyword (exercise)
	#3	Keyword (physiotherapy)
	#4	Keyword (cobb angle)
	#5	#1 AND #2 OR #3 AND #4
	Limits	Clinical trials and English language
PubMed/15 January 2022	#1	ALL Fields (adolescent idiopathic scoliosis)
	#2	ALL Fields (exercise)
	#3	ALL Fields (physiotherapy)
	#4	ALL Fields (cobb angle)
	#5	#1 AND #2 OR #3 AND #4
	Limits	Randomized controlled trials and English language
PEDro/15 January 2022	#1	Title or Abstract (adolescent idiopathic scoliosis)
	#2	Title or Abstract (exercise)
	#3	Title or Abstract (physiotherapy)
	#4	Title or Abstract (cobb angle)
	#5	#1 AND #2 OR #3 AND #4
	Limits	Clinical trials and English language
ScienceDirect (Elsevier)/15 January 2022	#1	Title or Abstract (adolescent idiopathic scoliosis)
	#2	Title or Abstract (exercise)
	#3	Title or Abstract (physiotherapy)
	#4	Title or Abstract (cobb angle)
	#5	#1 AND #2 OR #3 AND #4
	Limits	Research articles and case reports and English language
EBSCOhost (GreenFILE)/15 January 2022	#1	TX All Text (adolescent idiopathic scoliosis)
	#2	TX All Text (exercise)
	#3	TX All Text (physiotherapy)
	#4	TX All Text (cobb)
	#5	#1 AND #2 OR #3 AND #4
	Limits	Randomized controlled trials and English language

Studies published in English; consisting of randomized controlled trials (RCTs); involving participants diagnosed with AIS from the age of 10 years and with a Cobb angle of more than 10 degrees; including physiotherapy intervention without any other related measures and comparison with placebo, control group (CG), or other physiotherapy interventions; and examining the Cobb angle were included in the study, whereas studies that are not in the English language, are not RCTs, were duplicates, do not examine the Cobb angle, and do not focus on physiotherapy exercises were excluded from the study.

The reviewer examined the selected studies and documented the following: author, the year of publication, study design, patient characteristics, exercise regimen information (type, length, dosage, and frequency of exercise), sample size, findings, and conclusions. To reach consensus, the reviewer analyzed the information along with two other analysts. The Cobb angle mean change score, which is the difference between the final and baseline scores, was derived from the included studies' means and standard deviations of the initial and final Cobb angle endpoint scores.

The reviewers assessed the methodological quality of the included studies using an 11-item PEDro scale to assess consistency [22]. As previously mentioned, studies with a score of eight were of excellent quality [23]. The Cochrane Collaboration approach was used to assess the likelihood of bias. Additionally, sequence generation, the concealment of allocation, blinding, the completeness of outcome results, and the lack of selective reporting of outcomes were evaluated. Each domain assessed the probability of bias as low, unknown, or high [24]. The quality of the evidence was determined for each meta-analysis using the Grading of Recommendations Assessment, Development, and Evaluation (GRADE) system [25]. This method involves reducing the quality of the proof from very high to moderate, low, or very low using specific variables. Biasness was rated as high, low, or ambiguous for each individual aspect in five categories (selection, performance, attrition, reporting, and others). For more information on the Cochrane risk of bias tool, see Table 2.

**Table 2:** Cochrane risk of bias of included studies. Y: yes; N: no; U: unclear; H: high; L: low.

Number	Study	Random sequence generation	Allocation concealment	Selective reporting	Other sources of bias	Blinding (participants and personnel)	Blinding (outcome assessment)	Incomplete outcome data	Conclusions
Scores		Y/N	Y/N	Y/N	Y/N	Y/N/U	Y/N	Y/N	H/L/U
1	Kumar et al. [26]	Y	Y	Y	Y	Y	Y	Y	L
2	Park et al. [27]	Y	Y	Y	Y	U	Y	Y	L
3	Shah et al. [28]	Y	Y	Y	Y	U	N	Y	U
4	Noll et al. [29]	Y	Y	Y	N	N	N	Y	H
5	Stark et al. [30]	Y	N	Y	Y	N	Y	Y	L
6	Schreiber et al. [31]	Y	N	Y	Y	U	N	Y	U
7	Wnuk et al. [32]	Y	N	Y	Y	Y	N	Y	L
8	Monticone et al. [33]	Y	Y	Y	Y	U	Y	Y	L
9	Trzcińska and Nowak [34]	Y	N	Y	Y	N	N	Y	H
10	Monticone et al. [35]	Y	Y	Y	N	N	Y	Y	U
11	Schreiber et al. [36]	Y	Y	Y	N	N	N	Y	H
12	Gür et al. [37]	Y	Y	Y	Y	N	N	Y	U
13	Gao et al. [38]	Y	Y	Y	Y	Y	N	Y	L
14	Zheng et al. [7]	Y	Y	Y	Y	Y	N	Y	L
15	Yagci and Yakut [39]	Y	Y	Y	Y	Y	N	Y	L
16	Kuru et al. [40]	Y	Y	Y	N	N	N	Y	H
17	Langensiepen et al. [41]	Y	Y	Y	N	N	N	Y	H
18	Alves de Araújo et al. [42]	Y	N	Y	N	N	N	Y	H
19	Abbott et al. [43]	Y	Y	Y	N	N	Y	Y	H
20	Atici et al. [44]	Y	N	Y	N	N	N	Y	H

Results

**Figure 1:**
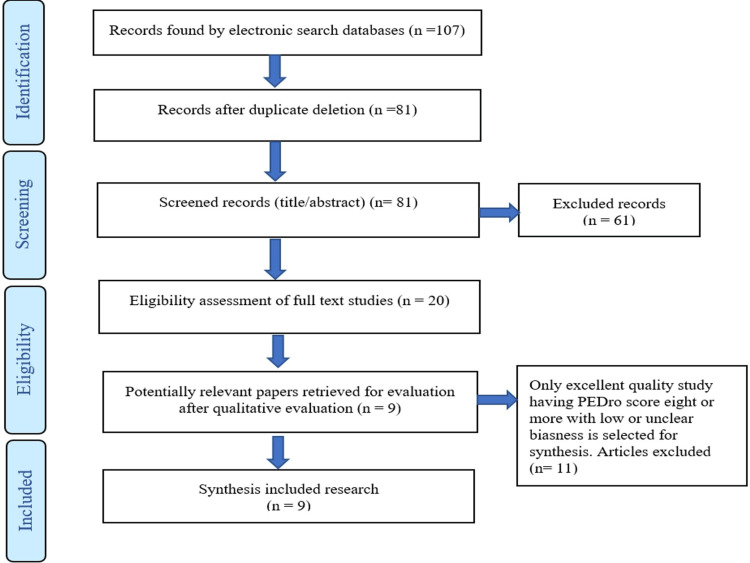
Flow diagram of search process and selection process. n: number of randomized controlled trials (RCTs); PEDro: Physiotherapy Evidence Database.

Electronic database searches produced 107 search papers, including 27 results from Cochrane Central Register of Controlled Trials, 28 results from EBSCOhost, two results from PEDro, 30 results from PubMed, and 19 results from ScienceDirect (Elsevier). Twenty studies were eventually chosen for quality assessment after 61 papers in which full text was not available, and 26 duplicate publications were removed in accordance with the inclusion and exclusion criteria. All of the articles were original research studies written in English that examined how exercise affected the Cobb angle (Figure 1).

All 20 studies were evaluated for quality (risk of bias) by using the PEDro score and Cochrane risk of bias. The lack of randomization, concealed allocation, and blinding were the most prevalent errors in the PEDro rankings (patients, therapists, or evaluators). The components of the PEDro scale that were most frequently employed were baseline comparability, follow-up, intention-to-treat analysis, measures of uncertainty, and between-group comparisons, which were evident in all articles. Table 3 shows the PEDro ratings of the selected studies.

**Table 3:** Summary of quality assessment on PEDro scale. Y: yes; N: no; PEDro: Physiotherapy Evidence Database.

Number	Study	Eligibility criteria	Random allocation	Concealed allocation	Baseline comparability	Blinded participants	Blinded therapists	Blinded assessors	Adequate follow-up	Intention-to-treat analysis	Between-group comparison	Point estimates and variability	Total score
Scores		Y/N	Y/N	Y/N	Y/N	Y/N	Y/N	Y/N	Y/N	Y/N	Y/N	Y/N	10
1	Kumar et al. [26]	Y	Y	Y	Y	Y	Y	Y	Y	Y	Y	Y	10
2	Park et al. [27]	Y	Y	Y	Y	Y	N	Y	Y	Y	Y	Y	9
3	Shah et al. [28]	Y	Y	Y	Y	Y	N	N	Y	Y	Y	Y	8
4	Noll et al. [29]	Y	Y	Y	Y	N	N	N	Y	Y	Y	Y	7
5	Stark et al. [30]	Y	Y	N	Y	Y	N	Y	Y	Y	Y	Y	8
6	Schreiber et al. [31]	Y	Y	N	Y	Y	N	N	Y	Y	Y	Y	7
7	Wnuk et al. [32]	Y	Y	N	Y	Y	N	N	Y	Y	Y	Y	7
8	Monticone et al. [33]	Y	Y	Y	Y	Y	N	Y	Y	Y	Y	Y	9
9	Trzcińska and Nowak [34]	Y	Y	N	Y	Y	N	N	Y	Y	Y	Y	7
10	Monticone et al. [35]	Y	Y	Y	Y	N	N	Y	Y	Y	Y	N	7
11	Schreiber et al. [36]	Y	Y	Y	Y	N	N	N	Y	Y	Y	Y	7
12	Gür et al. [37]	Y	Y	Y	Y	Y	N	N	Y	Y	Y	Y	8
13	Gao et al. [38]	Y	Y	Y	Y	Y	Y	N	Y	Y	Y	Y	9
14	Zheng et al. [7]	Y	Y	Y	Y	Y	Y	N	Y	Y	Y	Y	9
15	Yagci and Yakut [39]	Y	Y	Y	Y	Y	Y	N	Y	Y	Y	Y	9
16	Kuru et al. [40]	Y	Y	Y	Y	N	N	N	Y	Y	Y	Y	7
17	Langensiepen et al. [41]	Y	Y	Y	Y	N	N	N	Y	Y	Y	Y	7
18	Alves de Araújo et al. [42]	Y	Y	N	Y	N	N	N	Y	Y	Y	Y	6
19	Abbott et al. [43]	Y	Y	Y	Y	N	N	Y	Y	Y	Y	Y	8
20	Atici et al. [44]	Y	Y	N	Y	N	N	N	Y	Y	Y	Y	6

According to an analysis of the overall risk of bias across eight research, the risk was low overall, high in seven of them, and undetermined in four. The probability of bias evaluation for the selected studies is shown in Table 2. The most frequent deficiencies are a lack of blindness, a lack of concealment, and insufficient random sequence production, but nine studies were considered of excellent quality (PEDro score of eight or more with low or unclear bias) that were included in this review, as described in Table 4.

**Table 4:** Potentially relevant articles that were included in the review. PEDro: Physiotherapy Evidence Database.

Number	Year	Researchers	Articles	PEDro score	Cochrane risk of biasness
1	2017	Kumar et al. [26]	Efficacy of task oriented exercise program based on ergonomics on Cobb's angle and pulmonary function improvement in adolescent idiopathic scoliosis- a randomized control trial	10	Low
2	2016	Park et al. [27]	The effect of a core exercise program on Cobb angle and back muscle activity in male students with functional scoliosis: a prospective, randomized, parallel-group, comparative study	9	Low
3	2014	Monticone et al. [33]	Active self-correction and task-oriented exercises reduce spinal deformity and improve quality of life in subjects with mild adolescent idiopathic scoliosis. Results of a randomised controlled trial	9	Low
4	2019	Gao et al. [38]	Could the clinical effectiveness be improved under the integration of orthotic intervention and scoliosis-specific exercise in managing adolescent idiopathic scoliosis?: a randomized controlled trial study	9	Low
5	2018	Zheng et al. [7]	Whether orthotic management and exercise are equally effective to the patients with adolescent idiopathic scoliosis in mainland China?: a randomized controlled trial study	9	Low
6	2019	Yagci and Yakut [39]	Core stabilization exercises versus scoliosis-specific exercises in moderate idiopathic scoliosis treatment	9	Low
7	2019	Shah et al. [28]	Ab1375-HPr effect of Schroth method and scientific exercise approach to scoliosis (SEAS) on the Cobb angle among the adolescent with idiopathic scoliosis a comparative study	8	Unclear
8	2017	Stark et al. [30]	Physiotherapy combined with mechano-stimulation in adolescent idiopathic scoliosis	8	Low
9	2017	Gür et al. [37]	The effectiveness of core stabilization exercise in adolescent idiopathic scoliosis: a randomized controlled trial	8	Unclear

The sample size ranged from 25 to 110 for the total study population, which had an average age of 10-25 years. The inclusion criteria for the participants with AIS in the selected studies were primarily based on the Cobb angle. Table 5 provides the description of participant attributes of the selected studies.

**Table 5:** PICO information of the studies included in the review. AIS: adolescent idiopathic scoliosis; TLSO: thoracic-lumbar-sacral orthosis; SEG: Schroth exercise group; PICO: population, intervention, control, and outcomes.

Number	Year of publication	Reference study	Sample size and characteristics		Intervention/task for N1	Intervention/task for N2	Time of intervention	Cobb angle				Conclusion
			Age	Participants in each group				Groups	Pre-intervention	Post-intervention	Mean change	
1	2017	Kumar et al. [26]	10-15	N1 (experimental group): 18 (seven females and 11 males); N2 (controlled group): 18 (eight females and 10 males)	Exercises recommended in control group plus task-oriented exercises	Spinal extension exercises, spinal strengthening of the convex side muscles, active self-correction, chest expansion exercises with emphasis on the concave side, and diaphragmatic breathing exercises	1 year	N1	12.61±1.81	6.83±1.72	−5.77±1.35	The therapy protocol has been of benefit to the AIS patients who have increased their Cobb angle dramatically relative to AIS patients who do not follow the specific exercise protocol prescribed to the control group
								N2	12.72±1.40	9.67±1.32	−3.05±0.80	
2	2016	Park et al. [27]	20-25	N1 (home-based exercise program): 25 males; N2 (community group-based exercise program): 28 males	Patients received an exercise video and were periodically telephoned by an instructor	Completed all exercises together in the gymnasium under direct supervision of an instructor	10 weeks	N1	9.12±2.26	7.07±3.01	−2.05±0.50	A 10-week core training program for both a home-based and a community-based organization lowered the Cobb angle
								N2	9.58±2.66	4.33±2.45	−5.25±0.11	
3	2014	Monticone et al. [33]	>10	N1 (experimental group): 55 (39 females and 16 males); N2 (controlled group): 55 (41 females and 14 males)	Active self-correction, task-oriented spinal exercises, and education	Traditional spinal exercises	1 year	N1	19.3±3.9	14.0±2.4	−5.3±0.6	A program of rehabilitation, including active self-adjusting, task-based exercises, and instruction, is useful for reducing the spinal deformation
								N2	19.2±2.5	20.9±2.2	1.7±0.3	
4	2019	Gao et al. [38]	>10	N1 (orthosis combined with exercise group): 25, left after six months = 22 (18 females and four males); N2 (only orthotic intervention group): 25, left after six months = 23 (18 females and five males)	Scientific exercise approach to scoliosis (SEAS), breathing exercise to improve lung capacity and rib mobilization, and TLSO	TLSO for 23 hours in a day and one hour for personal hygiene	6 months	N1	29.13±4.32	24.26±1.96	−4.87±0.24	A Cobb angle adjustment was best provided in patients with idiopathic scoliosis compared with an orthotic treatment only through orthotic intervention in conjunction with scoliosis-specific training
								N2	28.64±3.91	26.59±3.57	−2.05±0.26	
5	2018	Zheng et al. [7]	10-17	N1 (exercise group): 30, left after six months = 24 (22 females and seven males); N2 (bracing group): 30, left after six months = 29 (19 females and five males)	SEAS	TLSO for 23 hours in a day and one hour for personal hygiene	1 year	N1	27.03±3.57	6 months: 25.45±3.60; 12 months: 24.79±4.36	6 months: −1.59±1.52; 12 months: −2.24±3.19	During intervention, the two treatments improved substantially with respect to spinal curvature parameters (Cobb angle and Cobb angle correction). Intergroup contrast findings also demonstrated that bracing in 12-month assessment was superior to capture correction in the Cobb angle
								N2	28.00±3.60	6 months: 25.25±3.58; 12 months: 22.13±4.78	6 months: −2.75±4.68; 12 months: −5.88±6.37	
6	2019	Yagci and Yakut [39]	>12	N1 (scientific exercise approach to scoliosis): 15 females; N2 (core stabilization {CS} group): 15 females	SEAS and TLSO for 23 hours in a day and one hour for personal hygiene	Core stabilization exercise and TLSO for 23 hours in a day and one hour for personal hygiene	4 months	N1	30.0±9.3	24.7±9.7	−5.3±2.2	Both treatment conditions, including CS bracing exercise or SEAS bracing exercise, decreased curve progression over the four-month span
								N2	27.6±8.0	21.4±7.1	−6.2±0.9	
7	2019	Shah et al. [28]	10-18	N1 (Schroth method of exercise group): 15 females/males; N2 (scientific exercise approach to scoliosis group {SEASG}): 15 females/males	The SEG performed Schroth exercise, five times a week for seven weeks	The SEASG performed scientific exercise, five times a week for seven weeks	7 weeks	N1	31.2±5.20	27.4±5.17	−3.8±0.03	The significant changes in the pre- and post-Cobb angle measurements were seen by both SEG and SEASG. Intergroup findings indicated that SEG was more successful than SEASG in changing the Cobb angle
								N2	31.33±5.26	29.4±5.9	−1.93±0.64	
8	2017	Stark et al. [30]	10-17	N1 (scoliosis-specific exercise {SSE} group): 15 females; N2 (SSE program on a side-alternating whole-body vibration {sWBV} platform group): 15 females	Regular scoliosis-specific exercises (SSE)	Home-based SSE program on a side-alternating whole-body vibration (sWBV) platform	6 months	N1	Data not available	Data not available	−0.3±3.7	Home-based SSE conducted on a six-month sWBV platform counteracts the progression of scoliosis in girls with AIS
								N2	Data not available	Data not available	−2.3±3.8	
9	2017	Gür et al. [37]	10-16	N1 (stabilization group): 12 (one male and 11 females); N2 (control group): 13 females	Core stabilization exercises	Traditional exercise programs and TLSO for 23 hours in a day and one hour for personal hygiene	10 weeks	N1	Thoracic: 35±11.82; lumbar: 29±8.35; total: 56.75±25.70	Thoracic: 28.45±11.86; lumbar: 23.63±10.39; total: 45.64±25.44	Thoracic: −6.73±2.69; lumbar: −5.13±5.49; total: −9.82±6.13	In the CS and control classes, the overall Cobb angle decreased by nine degrees and two degrees on average, respectively. Comparisons of the Cobb angle between the groups showed substantially higher increases in the stabilization group than in the control group
								N2	Thoracic: 31.42±6.97; lumbar: 34.33±2.2; total: 60.69±17.75	Thoracic: 33.88±7.34; lumbar: 32.63±10.2; total: 59.11±19.99	Thoracic: 0.63±4.34; lumbar: −1.75±3.45; total: −2.11±6.31	

The Cobb angle was measured using radiographic techniques in each study, and the control group underwent standard treatment or a regular exercise regimen. Table 6 displays the training regimens and outcomes for the exercise, control, and other intervention groups in the included studies.

**Table 6:** Analysis of the effect of exercise on Cobb angle in the included studies. SSE: scoliosis-specific exercise; sWBV: side-alternating whole-body vibration.

Number	Reference study	Cobb angle				
		Groups	Pre-intervention	Post-intervention	Mean change	P-value
1	Kumar et al., 2017 [26]	N1 (experimental group)	12.61±1.81	6.83±1.72	−5.77±1.35	<0.001*
		N2 (controlled group)	12.72±1.40	9.67±1.32	−3.05±0.80	<0.001*
2	Park et al., 2016 [27]	N1 (home-based exercise program)	9.12±2.26	7.07±3.01	−2.05±0.50	<0.001*
		N2 (community group-based exercise program)	9.58±2.66	4.33±2.45	−5.25±0.11	<0.001*
3	Monticone et al., 2014 [33]	N1 (experimental group)	19.3±3.9	14.0±2.4	−5.3±0.6	<0.001*
		N2 (controlled group)	19.2±2.5	20.9±2.2	1.7±0.3	<0.001*
4	Gao et al., 2019 [38]	N1 (orthosis combined with exercise group)	29.13±4.32	24.26±1.96	−4.87±0.24	<0.001*
		N2 (only orthotic intervention group)	28.64±3.91	26.59±3.57	−2.05±0.26	0.053
5	Zheng et al., 2018 [7]	N1 (exercise group)	27.03±3.57	6 months: 25.45±3.60; 12 months: 24.79±4.36	6 months: −1.59±1.52; 12 months: −2.24±3.19	6 months: 0.122; 12 months: 0.03*
		N2 (bracing group)	28.00±3.60	6 months: 25.25±3.58; 12 months: 22.13±4.78	6 months: −2.75±4.68; 12 months: −5.88±6.37	6 months: 0.062; 12 months: <0.001*
6	Yagci and Yakut, 2019 [39]	N1 (scientific exercises approach to scoliosis)	30.0±9.3	24.7±9.7	−5.3±2.2	<0.001*
		N2 (core stabilization group)	27.6±8.0	21.4±7.1	−6.2±0.9	<0.001*
7	Shah et al., 2019 [28]	N1 (Schroth method of exercise group)	31.2±5.20	27.4±5.17	−3.8±0.03	<0.001*
		N2 (scientific exercise approach to scoliosis group)	31.33±5.26	29.4±5.9	−1.93±0.64	<0.001*
8	Stark et al., 2017 [30]	N1 (SSE group)	Data not available	Data not available	−0.3±3.7	>0.05
		N2 (SSE program on a sWBV platform group)	Data not available	Data not available	−2.3±3.8	<0.001*
9	Gür et al., 2017 [37]	N1 (stabilization group)	Thoracic: 35±11.82; lumbar: 29±8.35; total: 56.75±25.70	Thoracic: 28.45±11.86; lumbar: 23.63±10.39; total: 45.64±25.44	Thoracic: −6.73±2.69; lumbar: −5.13±5.49; total: −9.82±6.13	Thoracic: <0.05; lumbar: <0.05; total: <0.05
		N2 (control group)	Thoracic: 31.42±6.97; lumbar: 34.33±2.2; total: 60.69±17.75	Thoracic: 33.88±7.34; lumbar: 32.63±10.2; total: 59.11±19.99	Thoracic: 0.63±4.34; lumbar: −1.75±3.45; total: −2.11±6.31	Thoracic: >0.05; lumbar: <0.05; total: >0.05

Discussion

This systematic review aimed to extract recently available evidence from 1 January 2010 to 15 January 2022 regarding the benefits of physiotherapy or exercise programs on the Cobb angle in AIS patients. The results suggest that physiotherapeutic interventions can lead to a decrease in the Cobb angle under different exercise or brace conditions. Still, the choice of physiotherapy intervention was somewhat arbitrary in all the studies. Nine studies were selected for review, which were considered to be of excellent quality (PEDro score of eight or more with low or unclear bias), and the analysis comprised a total of 411 participants (133 males and 278 females).

The PEDro score was nine out of 10 in five tests and eight in three studies. In the overall Cochrane measurement, seven trials showed a low risk of bias, and two had undetermined risks of bias since most of the investigations were not carried out by blinded therapists or assessors (Table 3).

The findings of this systematic study provide low-to-high-quality evidence in support of a medium-effect intervention in order to reduce Cobb angle in AIS patients. In a randomized controlled trial (RCT), 36 males and females between the ages of 10 and 15 were divided into two groups: an experimental group and a control group consisting of 18 participants each. While the subjects in the experimental group were accompanied for a year by task-oriented ergonomic exercises in addition to traditional exercises, those in the control group underwent spinal reinforcement, active self-correction, and breathing exercises. The Cobb angle decreased significantly before and after the intervention in both groups and was significantly higher in the task-oriented exercise groups than in the control group. This shows that exercise helps to avoid curve formation and reduces abnormalities in AIS [26].

Similarly, a trial recruited 53 patients in their sample involving only males aged 20-25 years that were randomly divided into two groups: a home-based exercise program group consisting of 25 patients and a community-based exercise program group consisting of 28 patients. The community-based group participated in all activities together, including the gymnasium, under the supervision of an instructor, whereas those in the home-based group received an activity video and phone calls from the instructor. The trial results demonstrated that Cobb angles were slightly smaller than the average in both groups during the 10-week exercise program, whereas the investigation predicted that the home-based exercise program would be less effective than the community-based group program, but there was no clinically relevant variation between them. This might be as a result of the trial's limited sample size or the lack of a control group. They also concluded that the patients who did not work out did not change, leading to ethical issues [27].

In another trial, 110 patients under the age of 10 (both males and females) were randomly assigned to two groups of 55 participants each: the experimental group, which underwent active self-correction, task-oriented spinal exercise, and education, and the control group, which followed conventional spinal exercise rehabilitation. Both groups held outpatient sessions once a week for 60 minutes and were also asked to continue the workout at home twice a week for one year in 30-minute sessions. The experimental group showed a Cobb angle decrease of more than five degrees, while the control group remained unchanged [33]. In patients with moderate AIS, the active self-correction and task-oriented exercise program were superior to standard Cobb angle reduction exercises, and its benefits persisted for at least a year after the completion of the intervention.

Additionally, 50 patients from an RCT that included both males and females under the age of 10 years were randomly split into two groups of 25 patients each. One group was given orthosis intervention (OI) in conjunction with exercise, and the other group was given OI alone. The study group conducted the scientific exercise approach to scoliosis (SEAS), and after six months of the intervention, patients in the orthosis paired with the exercise group were found to correct the Cobb angle more effectively than those in the orthosis control group [38]. In scoliosis management, different orthotic designs are available, varying in terms of the construction process, rigidity, system of action, and field of action. The Boston brace is an independently fitted orthosis with correction pads positioned on the curve convexity and comfort points, preventing development by adding a three-point spinal curvature pressure, which was discovered by Kalichman et al. [45]. However, it is important not to overlook the weakening of the back muscle caused by OI, as it is necessary to preserve spinal alignment and stabilize the posture of the body [46].

Similarly, in a trial consisting of 60 patients (both males and females) aged between 10 and 17 years, patients were randomly divided into an exercise group and an OI group consisting of 30 patients each. The SEAS was conducted by the study group, in which after 12 months of intervention, both approaches showed a substantial change in the Cobb angle. Bracing outperformed Cobb angle correction after a 12-month evaluation, based on the results of the intergroup comparison. However, there is no doubt that bracing has proven to be beneficial in preventing the deformity's progression and minimizing the need for surgery. Furthermore, because of their enhanced mental health, the patients in the exercise group were believed to have a more positive outlook on their physical appearance. On the other hand, owing to the tension of the bracing, the perception of the bracing community may be distorted [7].

A recent RCT enrolled a total of 30 patients, consisting of only females aged between 10 and 17 years, who were randomly assigned to the SEAS group and the core stabilization (CS) group consisting of 15 patients each. Along with the exercises, spinal braces were introduced in both groups. The patients were instructed to wear the brace for 23 hours daily and remove it only after exercise and for one hour each day while doing personal grooming. Both SEAS and CS exercise sessions lasted for 40 hours. In conclusion, the findings indicate that all patients had decreased thoracic and lumbar Cobb angles of the scoliotic curve and that both SEAS bracing and CS bracing exercises were effective in limiting curve development over a four-month period. These findings demonstrate that both treatment regimens were effective in preventing the formation of curves in individuals with mild curve AIS and that their effects on the Cobb angle were equivalent. In patients with AIS who were given only exercise recommendations, Negrini et al. found that SEAS exercises were more beneficial than conventional physiotherapy and also observed that SEAS was able to minimize corrective losses while wearing a brace in cases of intermediate curves [13]. The main target of scoliosis therapy is to enhance cosmetic appearance as the cosmetic deformity was greatly increased in both groups in this study. After four months of exercise and bracing therapy, a significant improvement in cosmetic deformity in both groups might be due to decreased curve amplitude and increased body symmetry [39].

Similarly, a trial enrolled 30 patients (both males and females) between 10 and 18 years of age, who were randomly allocated to the SEAS group and the Schroth exercise group (SEG) involving 15 patients each. Both the groups completed their respective exercises, five days a week for seven weeks, and after seven months of intervention, both the SEG and SEAS group showed significant improvements in pre- and post-Cobb angle measurements. Intergroup findings indicated that Schroth could be more effective than SEAS in modifying the mild-to-severe Cobb angle in adolescent idiopathic scoliosis [28].

Another study included 30 female patients with scoliosis aged between 10 and 17 years who received home-based programs and were randomly assigned to two groups: one received scoliosis-specific exercises (SSE) on a side-alternating whole-body vibration (sWBV) platform, while the other received regular SSE. Using magnetic resonance imaging (MRI), the Cobb angle was measured at the beginning and after six months along with reporting the onset of menarche in a subgroup study. This study concluded that home-based SSE performed on the sWBV platform for six months prevents females with AIS from developing scoliosis, especially before menarche. The discrepancy between the two groups was statistically significant, and the clinical significance of the main curve was as follows: 20% of the sWBV increased by more than or equal to five, 75% was stable, and 5% decreased. The test group improved by 0%, stabilized by 8%, and decreased by 11%. The subgroup study also revealed the most scientifically important improvement in the before-menarche subgroup [30].

In a previous trial that enrolled 25 patients (one male and 24 females) between the ages of 10 and 16 years, patients were randomly assigned to the stabilization group (SG) (n=12) and the control group (CG) (n=13). The CG underwent traditional scoliosis exercise programs, which included breathing exercises, posture preparation, spinal stability exercises, stretching exercises for the affected muscles (especially for the concave side of the curve), and general strengthening exercises for the affected muscles (especially for the convex side of the curve). The SG underwent central stabilization exercises in addition to the standard recovery protocol. The analysis concluded that the overall Cobb angle decreased by an average of nine degrees and two degrees in SG and CG, respectively. In contrast, intergroup analysis of the Cobb angle demonstrated a far greater change in the SG than in the CG [37].

In the current systematic review, four studies compared SEAS exercises with CG or other intervention groups and stated that SEAS exercises were more beneficial in controlling conditions for the reduction of spinal deformities and the development of scoliosis. This review included five more trials that evaluated five different exercise regimes with standard spinal exercises, with most of these experimental studies showing a substantial reduction in Cobb angle. However, this study had significant drawbacks, including the inclusion criteria not being explicitly stated in the included studies and the fact that the majority of the included trials were not randomized. Furthermore, the latest published literature has revealed substantial limits on the absence of blinding, masked allocations, and differences in exercise protocols. Additionally, various forms of exercise have different intensities with different outcomes, and the existence of heterogeneity in exercise procedures prevents definitive conclusions. For instance, the overall period of intervention varied from 10 weeks to 12 months, and the sample size of the included studies ranged from 25 to 110. Another drawback of the current study is that it only included studies written in English, which may have biased the selection process, and the majority of the studies did not specify which exercises were included in the standard protocol.

## Conclusions

Idiopathic scoliosis treatment is complex, and therefore, a thorough analysis of the deformity and each patient's clinical picture should be the key point in the conservative treatment of AIS. In terms of the efficiency of PSSE, it is found that SEAS exercises are more beneficial at improving the Cobb angle and preventing aggravation from brace wear than standard physical therapy. In addition, Schroth method offers useful information for treating and preventing scoliosis. Although the three approaches, SEAS, Schroth, and CS, seem to address the major bulges, they also improve the quality of treatment and life of the patients by stabilizing the results and reducing the progress of scoliosis. In conclusion, the severity of this deformation should, however, serve as yet another motivator for the therapeutic regimen and clinical judgment of therapists rather than a treatment-blocking factor, according to scientifically based knowledge and its implementation in the treatment of AIS.

According to the findings of the included studies, a therapeutic regimen is more effective than controls in lowering the Cobb angle in patients with AIS. Additionally, it demonstrates that bracing while exercising will produce superior Cobb angle reduction benefits than exercise alone. However, the validity of these findings is constrained by the variability of the exercise protocols and inadequate methodological quality.
